# Anosognosia in Alzheimer's disease: the interplay between resting state Default Mode Network and Salience Network

**DOI:** 10.1002/alz.087960

**Published:** 2025-01-03

**Authors:** Manuela Tondelli, Daniela Ballotta, Chiara Carbone, Riccardo Maramotti, Annalisa Chiari, Giovanna Zamboni

**Affiliations:** ^1^ Neurologia, Azienda Ospedaliero Universitaria di Modena, Modena Italy; ^2^ Università di Modena e Reggio Emilia, Modena Italy

## Abstract

**Background:**

Recent evidence suggests that unawareness in Alzheimer’s disease (AD) continuum can be explained by a failure of the connections between brain regions involved in accessing and monitoring self and other information. It has been demonstrated that AD patients with anosognosia have reduced network connectivity in the default mode network (DMN); in addition, stronger connectivity of bilateral anterior cingulate cortex (ACC) was showed to be associated with anosognosia in prodromal AD suggesting a possible role of this region in mechanisms of “adaptation” to anosognosia early in the disease. Therefore, we hypothesized that anosognosia in AD could be associated with an imbalance between the activity of the DMN and the salience network (SN) detectable using resting state functional magnetic resonance imaging (fMRI).

**Methods:**

Sixty patients with MCI and AD dementia underwent fMRI and neuropsychological assessment including the Anosognosia Questionnaire Dementia (AQ‐D), a measure of anosognosia based on a discrepancy score between the patient’s and carer’s judgments. Independent component analysis was applied and: i) correlation analyses between the AQ‐D score and functional connectivity in SN and DMN, and ii) comparison analyses of functional connectivity in DMN and SN between aware or unaware patients were performed.

**Results:**

AQ‐D scores negatively correlated with intrinsic functional connectivity within the DMN in the retrosplenial cortex and precuneus, irrespective of cognitive impairment stage and age. We also found that unaware patients had higher connectivity within the SN in the anterior cingulate cortex compared to aware patients.

**Conclusion:**

in patients with MCI and AD dementia, higher degrees of anosognosia are associated with lower functional connectivity within the DMN in the retrosplenial cortex and higher functional connectivity within the SN in the anterior cingulate cortex. This suggests that DMN and salience network might interplay in anosognosia expression in the AD continuum.

